# Learning effects in visual grading assessment of model-based reconstruction algorithms in abdominal Computed Tomography

**DOI:** 10.1016/j.ejro.2023.100490

**Published:** 2023-05-06

**Authors:** Bharti Kataria, Jenny Öman, Michael Sandborg, Örjan Smedby

**Affiliations:** a Department of Radiology, Linköping University, Linköping, Sweden; b Department of Health, Medicine & Caring Sciences, Linköping University, Linköping, Sweden; c Center for Medical Image Science & Visualization (CMIV), Linköping University, Linköping, Sweden; d Department of Medical Physics, Linköping University, Linköping, Sweden; e Department of Biomedical Engineering and Health Systems (MTH), KTH Royal Institute of Technology, Stockholm, Sweden

**Keywords:** Computed tomography, Abdominal, Image quality, Learning effect, Visual grading, Perception

## Abstract

**Objectives:**

Images reconstructed with higher strengths of iterative reconstruction algorithms may impair radiologists’ subjective perception and diagnostic performance due to changes in the amplitude of different spatial frequencies of noise. The aim of the present study was to ascertain if radiologists can learn to adapt to the unusual appearance of images produced by higher strengths of Advanced modeled iterative reconstruction algorithm (ADMIRE).

**Methods:**

Two previously published studies evaluated the performance of ADMIRE in non-contrast and contrast-enhanced abdominal CT. Images from 25 (first material) and 50 (second material) patients, were reconstructed with ADMIRE strengths 3, 5 (AD3, AD5) and filtered back projection (FBP). Radiologists assessed the images using image criteria from the European guidelines for quality criteria in CT. To ascertain if there was a learning effect, new analyses of data from the two studies was performed by introducing a time variable in the mixed-effects ordinal logistic regression model.

**Results:**

In both materials, a significant negative attitude to ADMIRE 5 at the beginning of the viewing was strengthened during the progress of the reviews for both liver parenchyma (first material: −0.70, *p <* 0.01, second material: −0.96, *p <* 0.001) and overall image quality (first material:−0.59, *p <* 0.05, second material::−1.26, *p <* 0.001). For ADMIRE 3, an early positive attitude for the algorithm was noted, with no significant change over time for all criteria except one (overall image quality), where a significant negative trend over time (−1.08, *p <* 0.001) was seen in the second material.

**Conclusions:**

With progression of reviews in both materials, an increasing dislike for ADMIRE 5 images was apparent for two image criteria. In this time perspective (weeks or months), no learning effect towards accepting the algorithm could be demonstrated.

## Highlights

• For the four criteria no positive attitude towards ADMIRE 5 could be demonstrated.
• An increasing dislike for ADMIRE 5 was apparent for two criteria.
• Initial positive evaluation of ADMIRE 5 for image noise was reinforced over time.

## 1 Introduction

The introduction of various new techniques and applications in diagnostic radiology often require a transition period for successful implementation in routine clinical practice. Image quality produced by new techniques/applications often diverge from the previous acceptable image quality and hence adaptation is necessary to acclimatise with interpreting, analysing and reporting of the new images to improve subjective perception and maintain diagnostic performance and confidence.

Transition from analogue to digital technique posed a dilemma, as the noise distribution in the digital images was different from that of the analogue images, thereby reducing reader diagnostic confidence. Considering the many advantages of digital imaging over analogue film-based imaging, adaptation to the new digital environment led to its successful implementation [1].

Training sessions are often the basis for learning to interpret images and improving the radiologist’s ability to identify abnormal lesions in mammograms [2] or enable new clinical applications such as Cardiac Computed Tomography Angiography (CCTA) [3], [4]. However, the learning process can be slow and may take more than one year [4].

The increasing number of Computed Tomography (CT) examinations has raised concerns about the associated radiation dose to the population. Improvements in computer hardware provided the necessary computational power allowing the renaissance of iterative reconstruction (IR) algorithms. IR algorithms (available in several strengths) have noise reduction properties that may improve image quality and allow for potential dose reductions [5]. However, with higher strengths of IR algorithms, changes in amplitude of different spatial frequencies of the noise lead to a blotchy, unappealing appearance of imaged anatomy and many studies mention an impairment in radiologists’ subjective perception of image quality and diagnostic performance [6], [7], [8], [9]. Increasing clinical experience with IR algorithms may possibly lead to a change in attitude, as was previously observed with digitalisation. However, there is little evidence whether such adaptation exists. Marin et al. [10] evaluated the effect of clinical experience and a particular IR algorithm adaptive statistical iterative reconstruction (ASiR, GE Healthcare, Milwaukee, WI) on the radiologists’ diagnostic performance, confidence and perception of subjective image quality during two reading sessions performed with a 3-year interval between sessions. They suggest preliminary evidence of a learning curve for images reconstructed with ASiR, but whether this observation applies to other IR algorithms is yet to be ascertained. One of the readers who participated in both studies perceived higher tolerance for the unappealing image appearance produced by AD5 towards the end of the review period in two previous studies from our group. This observation initiated further analyses of data from the two studies [6], [8].

We had hypothesised that there is a change in readers´ assessment towards a more positive attitude to the Advanced modeled iterative reconstruction algorithm (ADMIRE, Siemens Healthineers, Germany), during the course of the review of images from two prior published studies [6], [8]. The aim of the current study was to test this hypothesis with new analyses of existing data by introducing a time variable in the ordinal logistic regression models.

## 2 Material and methods

Two earlier published studies [6], [8] approved by the regional ethical board compared image quality in abdominal CT at varying milliampere-seconds (mAs) values, reconstruction algorithms and slice thickness in the same patient to ascertain potential dose reductions by evaluating the performance of ADMIRE strengths 3 (AD3) and 5 (AD5). Informed written consent was obtained from 25 patients (first material) and 50 patients (second material) undergoing clinical abdominal CT performed on a dual source CT scanner, SOMATOM Force (Siemens Healthineers) in the experimental mode to obtain three dose levels per patient with one acquisition by splitting the dose between the two x-ray tubes. In the first material, all 25 examinations were non-enhanced and the second material consisted of two groups, of which 25 examinations were contrast-enhanced and 25 were non-enhanced. Contrast-enhanced examinations were performed in the venous phase using Ultravist 370 mgI/mL with an individual flow-rate and volume depending on patient demographics, calculated using OMNIVIS (GE healthcare). Due to the shorter length of one of the two image detectors, only patients with a Body Mass Index (BMI) of < 30 were included. Study subject demographics, acquisition parameters and study design characteristics including the pairwise comparisons and regression analyses performed have been previously reported and a detailed description is available in the published studies [6], [8]. For an overview of the same, please refer to the supplementary material provided with this paper. The image criteria from the European guidelines for quality criteria in CT [11] were used to suit the purpose of each study. In both studies the images were anonymised and displayed in random order on PACS workstations and simultaneous pairwise comparison of multi-planar reconstruction (MPR) (first study [6]) and axial (second study [8]) was performed by random assignment of images to either the right or left monitor.

In the first study [6], four readers rated MPR image pairs (axial, coronal and sagittal) reconstructed with AD3 and AD5 at 2 mAs values (42mAs and 98mAs) and slice thicknesses of 1 mm, 2 mm and 3 mm during the time interval February to May 2018.

In the second study [8], five readers independently rated axial image pairs reconstructed with filtered back projection (FBP), AD3 and AD5 at 3 mAs values (42, 98 and 140 mAs) during the time interval from March to June 2016. The selected assessment criteria for each of the studies are presented in Table 1. Each reader assessed 500 (first study) or 600 (second study) image pairs by applying grading scores based on a Likert-type scale as follows:

**Table 1:** Assessment criteria selected for first Kataria et al. [6] and second Kataria et al. [8] materials.

Criteria (Kataria et al., 2020)	Criteria (Kataria et al., 2018)
C1: Visually sharp reproduction of liver parenchyma	C1: Visually sharp reproduction of liver parenchyma
C2: Visually sharp reproduction of pancreas contour	C2: Visually sharp reproduction of pancreas contour
C3: Visually sharp reproduction of kidneys and proximal ureters	C3: Visually sharp reproduction of kidneys and proximal ureters
C4: Reproduction of contours of lymph nodes < 15 mm in diameter	C4: Reproduction of contours of lymph nodes < 15 mm in diameter
C5: Overall image quality for diagnostic purposes	C5: Image noise not affecting interpretation
	C6: Overall image quality for diagnostic purposes

− 2 images on the left monitor are better than images on the right monitor.

− 1 images on the left monitor are probably better than images on the right monitor.

0 Images on the left and right monitor are equivalent.

+ 1 images on the right monitor are probably better than images on the left monitor.

+ 2 images on the right monitor are better than images on the left monitor.

For the current study, additional data concerning the time order of ratings were also included in the analyses. The sequential numbering of observations was rescaled to a continuous time variable ranging from 0 (start of first viewing session) to 1(end of last viewing session). Three readers participated in both studies and all readers had a minimum of 2–3 years of working experience with IR algorithms at the start of the evaluation.

### 2.1 Statistical analysis

As the visual grading scores are defined on an ordinal scale, and there are dependencies between scores from each observer and each patient, an appropriate statistical method for such data must be used. In earlier publications, we have proposed statistical methods dedicated for situations like these [12], [13], [14].

In both parts of this study, the analysis was performed with Visual Grading Regression (VGR) [12], i.e. an ordinal logistic regression model, into which an additional time variable was introduced. For the first material [6], a mixed-effects ordinal logistic regression model was defined with reviewer and patient identity as random effects [14], a continuous fixed effect for the logarithm of the mAs (log mAs) and two categorical fixed effects for MPR slice thickness (2 mm and 3 mm, with 1 mm as the baseline). The model also included one fixed effect for reconstruction algorithm (AD5; categorical, using AD3 as the baseline) and, finally, one for the interaction between AD5 and time (continuous), representing a tendency towards higher or lower assessment scores for AD5 with time.

Also for the second material [8], the mixed-effects ordinal logistic regression included reviewer and patient identity as random effects. Since the results of the previous study [8] indicated that the relationship between mAs and image quality in this material could not be modelled as one simple relationship, in this case the mAs was modelled with two separate categorical fixed effects (for 98 mAs and 140 mAs, treating 42 mAs as the baseline category). Two additional categorical fixed effects represent reconstruction algorithms (AD3 and AD5, with FBP as the baseline), and finally the interaction terms between AD3 and time, and AD5 and time. An additional analysis, only including comparisons involving AD3 and AD5, used instead AD3 as the baseline category.

With variables coded as described above, the value of the regression coefficient for AD5 represents the effect of the new reconstruction algorithm at the beginning of the first viewing session. A positive or negative value of the regression coefficient for the interaction term indicates that the scores for AD5 relative to AD3 increase or decrease, respectively, during the course of the viewing.

All statistical calculations were performed in R, version 4.0.2 (https://www.r-project.org), using the *clmm* command [15]. The goodness of fit was reported using McFadden’s pseudo *R*^2^ which provides an indication of the goodness of fit analogous to the *R*^2^ of linear regression models [16].

## 3 Results

**Fig. 1:**
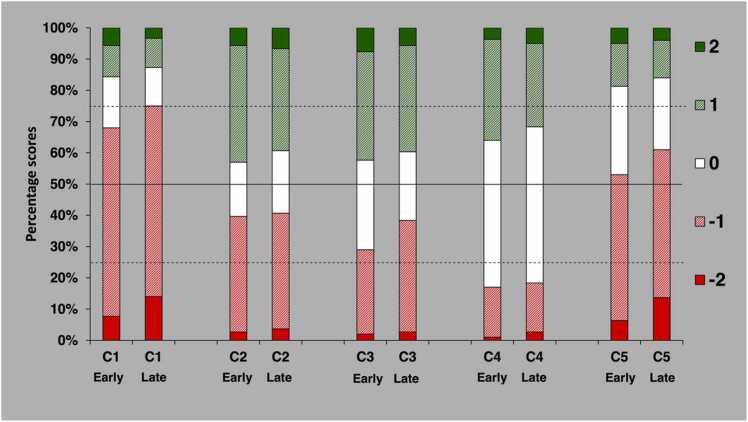
Distribution of scores comparing ADMIRE strength 5(AD5) to strength 3(AD3) in the first material [6], based on 600 comparisons per criterion. Score 2 indicates that AD5 was rated as superior to AD3, score 1 that AD5 was rated as probably superior to AD3, score 0 that the alternatives were rated as equivalent, score − 1 that AD5 was rated as probably inferior to AD3, and score − 2 that AD5 was rated as inferior to AD3. Early refers to the first 250 comparisons by each reader and late to the last 250 comparisons by each reader. The solid horizontal line represents the median and the dashed lines the upper and lower quartile.

The distribution of scores comparing AD5 to AD3 in the first material are shown in Fig. 1, where the material has been split in two halves corresponding to the first 250 (early) and last 250 (late) comparisons for each reader.

For the first image quality criterion (C1: liver parenchyma) and fifth criterion (C5: overall image quality), there was in the early comparisons a predominance of negative scores for AD5, which was more pronounced in the late comparisons, whereas no visually obvious change was found for the remaining criteria (C2: pancreas contour, C3: kidneys & ureters and C4: lymph nodes).

In the regression analysis (Table 2), regression coefficients and corresponding significance levels were obtained for the continuous effect representing mAs and for the two categorical effects representing slice thickness of 2 mm and 3 mm (both relative to the reference category 1 mm), as well as for the reconstruction algorithm (AD5, relative to AD3). The interaction term (AD5 × time) represents the change in preference between algorithms over time. The effects of mAs and slice-thickness variables were strongly significant for all image quality criteria. The values of these coefficients were identical to those obtained in the original analysis [6]. For the first criterion (C1: liver parenchyma), there was a strongly significant negative effect of AD5 and its interaction with time, indicating that the initially negative evaluation of AD5 relative to AD3 became even stronger during the viewing sessions. Similar findings were observed for the fifth criterion (C5: overall image quality). For the third criterion (C3: kidneys & proximal ureters), there was a significant positive coefficient for AD5, but a significant negative coefficient of similar size for the interaction term, indicating a positive attitude to AD5 at the beginning of the reading session, which vanished during the review. For the remaining criteria (C2: pancreas contour and C4: lymph nodes), the interaction term had no significant effect.

**Table 2:** Visual Grading Regression (VGR) based on 2000 comparisons of mAs, reconstruction algorithms ADMIRE 3(AD3) and ADMIRE 5(AD5) and slice thickness for the first material [6]. The reference algorithm is AD3.

**Criteria**	Regression coefficients					Whole-model *p* value	McFadden pseudo *R*^2^
	log (mAs)	Slice 2 mm	Slice 3 mm	AD5	AD5 × time		
**C1. Liver parenchyma**	1.25***	0.49***	0.53***	–1.00***	–0.70**	< 0.0001	0.083
**C2. Pancreas contours**	1.75***	0.47***	0.50***	0.16°	–0.22°	< 0.0001	0.059
**C3. Kidneys and proximal ureters**	1.78***	0.55***	0.55***	0.49**	–0.56*	< 0.0001	0.064
**C4. Lymph nodes ≤ 15 mm**	1.55***	0.51***	0.49***	0.65***	–0.34°	< 0.0001	0.061
**C5. Overall image quality**	1.65***	0.71***	0.87***	–0.56***	–0.59*	< 0.0001	0.077

*** ) *p* < 0.001, **) *p* < 0.01, * ) *p* < 0.05, °) not significant

**Fig. 2:**
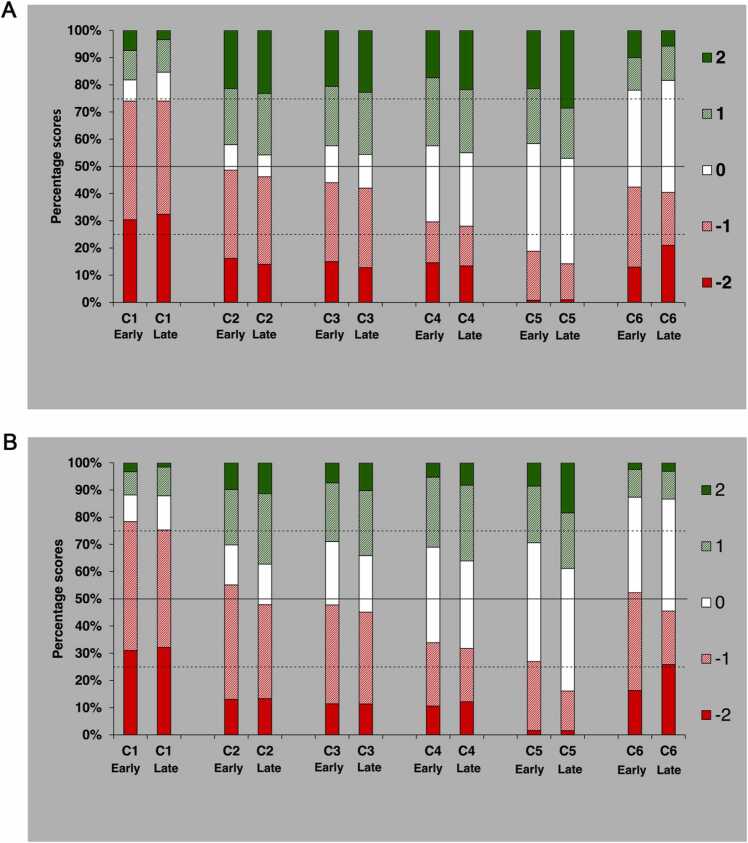
A. Distribution of scores comparing AD5 to the alternative (FBP or AD3) in the second material [8] based on 1000 comparisons per criterion. Score 2 indicates that AD5 was rated as superior to the alternative, score 1 that AD5 was rated as probably superior to the alternative, score 0 that the alternatives were rated as equivalent, score –1 that AD5 was rated as probably inferior to the alternative, and score –2 that AD5 was rated as inferior to the alternative. Early refers to the first 300 comparisons by each reader and late to the last 300 comparisons by each reader. The solid horizontal line represents the median and the dashed lines the upper and lower quartile. B. Distribution of scores comparing ADMIRE strength 5(AD5) to strength 3(AD3) in the second material [8] based on 500 comparisons per criterion. Score 2 indicates that AD5 was rated as superior to AD3, score 1 that AD5 was rated as probably superior to AD3, score 0 that the alternatives were rated as equivalent, score − 1 that AD5 was rated as probably inferior to AD3, and score − 2 that AD5 was rated as inferior to AD3. Early refers to the first 150 comparisons by each reader and late to the last 150 comparisons by each reader. The solid horizontal line represents the median and the dashed lines the upper and lower quartile.

In the second material, an analysis of all the scores comparing AD5 to either AD3 or FBP (Fig. 2A) was performed. Here, predominantly negative scores for AD5 are seen with C1 (liver parenchyma) and C6 (overall image quality). For C5 (image noise), on the other hand, the scores favourable for AD5 dominate. Both these patterns are slightly more apparent in the late assessments than in the early ones.

The regression analysis of the same material is presented in Table 3A, where both AD3 and AD5 are compared to FBP. The image quality of AD3 was rated as significantly superior to that of FBP, with no significant change over time for all criteria except C6 (overall image quality) for which, the initially positive attitude diminished over time. Still at the end of the review period, a clearly positive effect remained (0.94 (2.02 −1.08 = 0.94), *p* < 0.001).

**Table 3A:** Visual Grading Regression (VGR) based on 3000 comparisons of mAs and reconstruction algorithms Filtered Back Projection (FBP), ADMIRE 3 (AD3) and ADMIRE 5 (AD5) in the second material [8]. The reference algorithm is FBP.

**Criteria**	Regression coefficients						Whole-model *p* value	McFadden pseudo *R*^2^
	98 mAs	140 mAs	AD3	AD3 × time	AD5	AD5 × time		
**C1. Liver parenchyma**	2.19^***^	2.38^***^	1.02^***^	–0.35°	0.43^**^	–0.96^***^	< 0.0001	0.178
**C2. Pancreas contours**	1.83^***^	1.94^***^	1.22^***^	0.27°	1.68^***^	0.34°	< 0.0001	0.118
**C3. Kidneys and proximal ureters**	2.01^***^	2.16^***^	1.20^***^	0.42°	1.98^***^	0.32°	< 0.0001	0.131
**C4. Lymph nodes ≤ 15 mm**	1.60^***^	1.68^***^	1.07^***^	0.31°	1.84^***^	0.30°	< 0.0001	0.102
**C5. Image noise**	2.21^***^	2.59^***^	2.00^***^	–0.17°	2.98^***^	0.92^***^	< 0.0001	0.198
**C6. Overall image quality**	2.31^***^	2.42^***^	2.02^***^	–1.08^***^	1.93^***^	–1.26^***^	< 0.0001	0.177

*** ) *p* < 0.001, **) *p* < 0.01, * ) *p* < 0.05, °) not significant

In addition, for AD5, a positive effect relative to FBP was seen initially for all criteria. For the reproduction of liver parenchyma (C1), this was overshadowed by a negative effect of the interaction between AD5 and time, so that at the end of the review period, there was a significant negative effect (−0.53 (0.43 −0.96 = −0.53), *p* < 0.001). For image noise (C5), the initial strong positive effect became even stronger during the review. For overall image quality (C6), the initial positive effect diminished significantly over time, but even at the end of the review, a significant positive effect remained (0.67 (1.93 −1.26 =0.67), *p* < 0.001).

There was a strongly significant effect of mAs (categorical variables) for all criteria at mAs values 98 and 140 mAs relative to 42 mAs. The effect of 140 mAs was rather similar to that of 98 mAs, in line with the original analysis where an increase in dose from 98 to 140 mAs did not lead to a corresponding increase in image quality [8].

To produce results somewhat analogous to those of the first material (comparing AD5 to AD3), an analysis involving AD5 and AD3, split in two halves corresponding to the early and late viewings for each reader (Fig. 2B), was carried out. Image criteria C1: liver parenchyma and C6: overall image quality, both show predominantly negative scores for AD5 with no obvious change in attitude between early and late assessments. However, for the remaining criteria C2- –C5 there was a tendency to slight increase in the number of favourable scores for AD5 when comparing early and late viewings.

In the regression analysis (Table 3B), the only criteria showing a significant change over time (significant coefficient for the interaction term) were C1 (liver parenchyma), for which initially negative AD5 scores were more pronounced with time, and C5 (image noise), where the initially positive AD5 scores were reinforced over time. For C3 (kidneys & ureters) and C4 (lymph nodes), the initial positive evaluation for AD5 did not significantly change during the review. For C2 (pancreas contour) and C6 (overall image quality), finally, there was no significant difference between the algorithms, nor a change over time.

**Table 3B:** Visual Grading Regression (VGR) based on 1500 comparisons of mAs and reconstruction algorithms ADMIRE 3(AD3) and 5 (AD5) for the second material [8]. The reference algorithm is AD3.

**Criteria**	Regression coefficients				Whole-model *p* value	McFadden pseudo *R*^2^
	98 mAs	140 mAs	AD5	AD5 × time		
**C1. Liver parenchyma**	2.06^***^	1.99^***^	–0.70^***^	–0.65*	< 0.0001	0.201
**C2. Pancreas contours**	1.87^***^	1.68^***^	0.29°	0.32°	< 0.0001	0.120
**C3. Kidneys and proximal ureters**	2.14^***^	1.97^***^	0.68^***^	–0.03°	< 0.0001	0.142
**C4. Lymph nodes ≤ 15 mm**	1.59^***^	1.35^***^	0.68^***^	–0.07°	< 0.0001	0.091
**C5. Image noise**	2.45^***^	2.74^***^	1.20^***^	1.50^***^	< 0.0001	0.187
**C6. Overall image quality**	2.31^***^	2.18^***^	0.05°	–0.40°	< 0.0001	0.199

*** ) *p* < 0.001, **) *p* < 0.01, * ) *p* < 0.05, °) not significant

## 4 Discussion

Iterative reconstruction techniques allow for substantial dose reductions while maintaining image quality [8], [9], [17], [18]. Unfamiliar visual appearance and image texture in IR images impairs the implementation of these algorithms clinically and limits dose optimisation. However, the common belief is that radiologists could adapt after an initial learning curve [10], [19], [20].

In this study, one of the reader’s perception regarding the unappealing image appearance produced by AD5 initiated further analyses of previous research. After participation in the review of both studies, their opinion was that they had developed a higher tolerance for the image quality produced by AD5 as the review sessions progressed. Even though the initial positive effect for overall image quality (C6) diminished significantly over time, at the end of the review, a significant positive effect remained (0.67, *p* < 0.001), which may explain the perception of higher tolerance. Although the readers were not aware of which reconstruction algorithms were compared in both materials, it was difficult to blind them to the discernible difference in image quality produced by AD5. This perception may continue to persist even with increasing reader experience [10].

Marin et al.’s [10] study provided preliminary evidence of a learning curve for images reconstructed with IR algorithms. The introduction of IR at our facility was met with scepticism and initially strength 1 of the Sinogram affirmed iterative reconstruction (SAFIRE, Siemens Healthineers, Germany) algorithm (which has a similar noise texture to FBP) was implemented clinically. When switching to ADMIRE in 2015, initially strength 1 was preferred, but a gradual increase to strength 3 (which is the clinical standard today) was judged possible. Adaptation to the higher strength of AD3 has been quite smooth, which has supported the belief that radiologists can learn to adapt with time and increasing experience. Similarly, clinical implementation of low-dose protocols has also been a slow process. With increasing experience and adaptation to the low-dose protocol, there has been an increase in the panorama of diseases (starting with kidney stones) that can be diagnosed with a low-dose abdominal CT, which is advantageous for groups of patients that are sensitive to radiation [21]. Marin et al. [10] also showed significant improvements in readers’ acceptance of image quality with time for 20%, 40% and 60% ASiR-FBP weighted datasets. In addition, their findings show that despite marginal improvement in diagnostic performance (detection of hyper-vascular lesions), the overall image quality scores for the 80% ASiR datasets remained similar or deteriorated slightly over time. Direct comparison of these results to those of the present study is not possible as the algorithms are based on different principles and different time spans for each of the studies.

In the present study, no obvious significant learning trend (adaptation trend) was observed for either AD3 or AD5 in majority of criteria assessed. A possible explanation could be that the review period of 4 months was short and that the actual time differences between early and late assessments for each reader are not known. For criterion liver parenchyma, when comparing AD3 to AD5, the negative attitude towards AD5 is consistent in both materials and has not changed over the two-year gap between the two studies. In the second material, the shorter experience of working with ADMIRE may have had bearing on the gradually diminishing positive attitude for criterion 6 (overall image quality) reflected by a significantly negative interaction term for AD3 (Table 3A). For AD5 in the same material, the predominantly negative attitudes for two of the criteria (liver parenchyma and overall image quality) were strengthened with time. This perception of overall image quality for the higher strength of IR is in concurrence with the results for the 80% ASiR-FBP weighted blending in [10].

In routine clinical work, the fear of making perceptual errors (which make up 60 ̶80% of all radiological errors) [22], may accentuate a more conservative attitude among radiologists. Perhaps this is a plausible explanation why radiologists are more cautious in adapting to the new image appearance. However, the preferred IR algorithm strength may depend on the diagnostic task. Martens et al. [18] evaluated optimal tube current and IR strength in two patient groups and found that higher strength (AD 4) was preferred when evaluating known liver lesions as compared to strength 3 in terms of subjective diagnostic image quality. Although lesion detection is a central parameter when determining optimal diagnostic image quality, such studies are time-consuming and require the ground truth (lesion) to be known and a larger sample size for generalisability of the results. Visual grading studies, on the other hand are relatively inexpensive and easy to perform to assess diagnostic image quality. The basic assumption in these studies is that if normal anatomy can be sharply reproduced, then the same applies to pathology.

As IR algorithms reduce noise more efficiently with increasing strength, it is not surprising that the only criterion with a significant positive interaction term for AD5 in the second material was C5 (image noise). Despite this positive effect, there was no change in attitude over time for the perceived overall image quality produced by AD5.

Since Marin et al.’s [10] assessments included determination of pathological findings, a washout period was necessary to address the issue of recall bias. As the present study assessed image quality based on certain anatomical criteria, a washout period may not be necessary. However, the present study was a subjective image quality study where radiologists focused on the task of image interpretation as opposed to a lesion detection study where radiologists use a deliberate search strategy and are more focused on searching [23]. It is possible that the lesion detection task is easier to learn than interpreting typically normal images that require a longer time to read [24].

As the reviews progressed, an increasing dislike for ADMIRE 5 images for at least two image criteria was apparent in both materials. In the time perspective of weeks or months, no learning effect towards the new algorithm could be demonstrated.

To assess the fit of the statistical model, we used McFadden’s Pseudo-*R*^2^. Similar to the *R*^2^ of the linear regression model, this parameter provides indication of goodness to fit of the logistic regression model with values of 0.2–0.4 indicating a good model fit [16]. However, low Pseudo-*R*^2^ values (especially in Table 2) are quite common in visual grading studies like the present one.

### 4.1 Limitations

Due to the retrospective nature of the present study based on two previous studies with different study designs, there are several limitations. Since the readers in both materials were provided a timeline (start and finish) for the assessments, the duration between sessions may have varied from few weeks to months. Thus, any changes noted are on the time scale of weeks or months rather than years. Similarly, review of the two materials was based on different reformations (MPR in first material and axial images in the second material) hence, comparison between the two materials was not possible and the effect of years of experience with ADMIRE/IR could not be pursued. As patients with BMI ≥ 30, who present a challenge concerning image quality, were excluded in both materials, our results are limited to a certain patient population size. In addition, due to different study designs and only three readers participating in both the studies, no within-reader (intra-reader) comparison could be performed on these data.

## 5 Conclusion

As the reviews in both materials progressed, an increasing dislike for ADMIRE 5 images was apparent at least for two image criteria (liver parenchyma and overall image quality). Thus, in the time perspective of weeks or months, no learning effect, reflected in a gradually more positive attitude towards the new algorithm, could be demonstrated.

## Ethical statement

All procedures performed in studies involving human participants were in accordance with the ethical standards of the institutional research committee (Linköping University) and the 1964 Helsinki declaration and its later amendments or comparable ethical standards. Both previously published studies were approved by the regional Ethical Review Board (EPN, Linköping; Diary number: EPN 2014–273–31 and EPN 2015–327–32) and informed consent was obtained before performing the CT examination.

## Funding statement

This work was supported by grants from Region Östergötland and the Medical Faculty at 10.13039/501100003945Linköping University; Avtal Läkarutbildning och Forskning (10.13039/100001424ALF) (RÖ-602731, RÖ-697941), Forskning och Utveckling (10.13039/501100009963FoU) (RÖ-724631, RÖ-620341), Regionfinansierade Forskning och Utbildning (RFoU) and the 10.13039/501100003793Swedish Heart-Lung Foundation (Grant: 2022-0492).

## CRediT authorship contribution statement

**Bharti Kataria:** Project administration, Funding acquisition, Data curation, Investigation, Conceptualization, Methodology, Validation, Visualization, Writing - original draft, Writing - review & editing. **Jenny Öman:** Conceptualisation, Methodology, Validation, Writing – review & editing, Visualisation. **Michael Sandborg:** Conceptualization, Funding acquisition, Methodology, Validation, Visualization, Writing - review & editing. **Örjan Smedby:** Conceptualization, Formal analysis, Funding acquisition, Investigation, Methodology, Validation, Visualization, Writing - review & editing.

## Declaration of Competing Interest

The authors declare that they have no known competing financial interests or personal relationships that could have appeared to influence the work reported in this paper.
